# Circular RNA profiling facilitates the diagnosis and prognostic monitoring of breast cancer: A pair‐wise meta‐analysis

**DOI:** 10.1002/jcla.23575

**Published:** 2020-11-07

**Authors:** Yanqing Ma, Xiaobin Niu, Sha Yan, Yuchun Liu, Ruihua Dong, Yongwei Li

**Affiliations:** ^1^ The Second Affiliated Hospital of Henan University of Traditional Chinese Medicine Zhengzhou Henan China

**Keywords:** breast cancer, circular RNA, diagnosis, meta‐analysis, prognosis

## Abstract

**Background:**

As circular RNAs (circRNAs) have been found to significantly involve in the onset and progression of multiple malignant tumors including breast cancer (BC), this study aims at evaluating the diagnostic and prognostic values of circRNAs in this malady.

**Methods:**

Available databases were thoroughly searched to collect studies on the diagnosis and/or prognosis of BC using circRNA profiling. The updated Quality Assessment of Diagnostic Accuracy Studies 2 (QUADAS‐2) tool and the Newcastle Ottawa Scale (NOS) were used to assess the underlying bias of included studies. Clinical characteristics of the studies were merged by the quantitative‐weighted integral method to obtain the combined effects.

**Results:**

Sixteen studies were included, comprising 2438 BC cases and 271 noncancerous controls. The expression signature covered 24 circRNAs (down‐regulated: circ‐VRK1, hsa_circ_0068033, hsa_circ_103110, hsa_circ_104689, and hsa_circ_104821; up‐regulated: circAGFG1, hsa_circ_0001785, hsa_circ_0108942, hsa_circ_0001785, hsa_circ_006054, hsa_circ_100219, hsa_circ_406697, circEPSTI1, circANKS1B, circGFRA1, circ_0103552, CDR1‐AS, has_circ_001569, hsa_circ_001783, circFBXL5, circ_0005230, circAGFG1, circ‐UBAP2, and circ_0006528). The sensitivity and specificity of circRNAs in distinguishing BC patients from noncancerous controls were 0.65 and 0.68, and the corresponding area under the curve was 0.66. Survival analysis revealed that patients showing highly expressed oncogenic circRNAs were associated with increased mortality risks of BC in overall survival (univariate analysis: hazard ratio [HR] = 3.30, *P* = .000; multivariate analysis: HR = 3.07, *P* = .000), and disease‐free survival (HR = 8.26, *P* = .000). Stratified analysis based on circRNA expression status and control type also showed robust results.

**Conclusions:**

Circular RNA profiling presents prominent diagnostic and prognostic values in BC, and can be rated as a promising tool facilitating its early diagnosis and survival.

## Graphical abstract

In this work, the diagnostic and prognostic significance of circular RNAs (circRNAs) in breast cancer was evaluated, including 16 studies in total, as well as the comparison between 2438 BC cases and 271 noncancerous controls. The meta‐analysis showed that the sensitivity and specificity of circRNAs in distinguishing BC patients from noncancerous controls were 0.65 and 0.68 separately, corresponding to an area under the curve of 0.66. According to the survival analysis revealed, it showed that there was a correlation relationship between patients with highly expression of oncogenic circRNAs and the increased mortality risks of BC in prognosis.

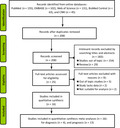

**Abbreviations**
AUC: area under the curve
BC: breast cancer
circRNA: circular RNA
DFS: disease‐free survival
FN: false negative
FP: false positive
GAPDH: reduced glyceraldehyde‐phosphate dehydrogenase
HR: hazard ratio
NOS: Newcastle Ottawa Scale
OS: overall survival
PFS: progression‐free survival
qRT‐PCR: quantitative reverse transcription‐polymerase chain reaction
QUADAS: Quality Assessment for Studies of Diagnostic Accuracy
RFS: relapse‐free survival
SEN: sensitivity
SPE: specificity
TN: true negative
TP: true positive

## 1 INTRODUCTION

Breast cancer (BC) tops the morbidity list among female malignancies, and the pace of its onset is accelerating year after year with the population becoming younger and younger.^1^, ^2^ As with the latest cancer statistics, the mortality of BC ranks the fourth among all female tumors.^3^ Studies have confirmed that family history, reproductive factors, sex hormone levels, oral contraceptives, and previous history of breast diseases are closely related to its occurrence and development.^4^, ^5^, ^6^ Exploring new molecular markers and therapeutic targets for BC are conducive to early diagnosis, more accurate prognostic prediction, and efficacy monitoring in the patients. At present, various factors restrict the early diagnosis of BC in clinic. Biopsy as an invasive method is poorly acceptable to patients, and its accuracy is subject to operators' own experience.^7^ Imaging examinations and routine blood tumor marker detection are currently not suitable for large‐scale screening for an early diagnosis due to their low sensitivity (SEN) and accuracy.^8^, ^9^ Therefore, finding effective, noninvasive, novel, and operable biomarker profiling is critical for the early diagnosis, prognosis, and treatment of BC.

Circular RNAs (CircRNAs) is a type of coding/noncoding RNA molecule with its 3ʹ and 5ʹ ends forming a covalently closed loop.^10^, ^11^ It is reported that circRNAs are widely expressed in mammalian cells and feature histocyte specificity (SPE), structural stability, and sequence conservation.^12^, ^13^ Studies have confirmed that circRNAs play roles in regulating gene transcription and expression via multiple pathways, and in physiological processes such as cell cycle and senescence.^14^, ^15^ Moreover, circRNAs are found to be essential in the onset and development of malignant tumors.^16^, ^17^ Given that circRNAs are insensitive to nucleases and more stable than ordinary linear RNA, they are expected to be new biomarkers for monitoring various cancers.^18^, ^19^ And circRNAs are intensively reported to have the potential of the early diagnosis and prognostic prediction of BC as novel molecular biomarkers.^20^, ^21^, ^22^, ^23^, ^24^, ^25^, ^26^, ^27^, ^28^, ^29^, ^30^, ^31^, ^32^, ^33^, ^34^, ^35^ However, the appraisals of their efficacy are commonly limited by the small sample size, high bias, and single‐center population of the already reported trials. Therefore, this study intends to systematically evaluate the efficacy of circRNAs in the diagnosis and prognostic prediction of BC through a quantitative meta‐analysis.

## 2 MATERIALS AND METHODS

### 2.1 Data search strategy

This study was designed and conducted in line with the PRISMA 2019.^36^ Two authors independently retrieved relevant studies in the online databases included PubMed, Embase, Web of Science, BioMed Central, and CNKI. Literature published in English, as of January 31, 2020, was searched. The following search terms were as follows: (“breast neoplasms [MeSH Terms]” OR “breast cancer” OR “breast carcinoma” OR “mammary cancer”) AND (“circular RNA [MeSH Terms]” OR “circRNA” OR “hsa circ”) AND (“diagnoses”, “diagnosis”, “SEN”, “SPE”, “ROC curve”, “area under the curve”, “AUC”) OR (“prognosis” OR “prognoses [MeSH Terms]” OR “survival [MeSH Terms]” OR “overall survival” OR “progression free survival” OR “disease free survival” OR “relapse free survival” OR “hazard ratio” OR “OS” OR “PFS” OR “DFS” OR “RFS” OR “HR”). Meanwhile, the authors manually searched for the references attached to the paper to prevent literature omission.

### 2.2 Inclusion and exclusion criteria

Inclusion criteria were defined as (a) case‐control studies that reporting the diagnostic accuracy or prognostic utility of single or parallel circRNAs in BC; (b) diagnostic studies providing data that could be directly or indirectly involved in a 2 × 2 contingency table, comprising true positives (TP), false positives (FP), false negatives (FN), and true negatives (TN); and (c) prognostic studies evaluating observation indicators with directly or indirectly provisions of HR values and 95% CIs, encompassing overall survival (OS), progression‐free survival (PFS) or disease‐free survival (DFS). The exclusion criteria were (a) studies with a sample size of less than 20; (b) or with insufficient data for statistical analysis; and (c) low‐quality studies and non‐English language articles.

### 2.3 Data extraction

Two authors independently screened the collected relevant studies and carefully extracted the following information: (a) basic clinical characteristics including the first author, publication time, study population, cohort size, control type, circRNA name, detection method, reference gene, cutoff value setting, AUC, follow‐up time, etc; and (b) data for statistical analysis incorporating TP, FP, FN, TN, SEN, SPE, HR values, and the corresponding 95% CIs. Patients with BC were considered the “case group,” and those with benign lesions or adjacent noncancer tissues or healthy individuals were deemed as the “control group or controls.”

### 2.4 Quality assessment

For diagnostic studies, the Quality Assessment of Diagnostic Accuracy Studies 2 (QUADAS‐2) tool was used to evaluate the quality of studies,^37^ and the evaluation consisted of 2 parts: bias evaluation and applicability. Specifically, the bias assessment included 4 domains: case selection, index test, golden standard, and flow and timing, and the first 3 domains were also assessed with respect to applicability. Each domain could be graded by 3 levels: low risk, high risk, and unknown, corresponding to 1 point, 0 point, and 0 point, respectively. When the total score was ≥4 points (out of 7 points), the quality of the study could be considered high. Case‐control studies were evaluated with the 8‐item Newcastle Ottawa Scale (NOS) scale,^38^ referring to study population selection, comparability, exposure evaluation, or outcome evaluation. A study with a total score of ≥5 points (out of 9 points) could be considered high quality.

### 2.5 Statistical analysis

Statistical analysis was performed by MetaDiSc 1.4 and Stata 12.0 software. The combined effect size indicators encompassed SEN, SPE, PLR, NLR, diagnostic odds ratio (DOR), AUC, HR, and 95% CI. The threshold effect was evaluated by Spearman's correlation coefficients, with a *P* < .05 considered statistically significant. The nonthreshold effect was evaluated by Cochran's *Q* test and *I* ^2^ test, and the statistical significance level was set at *P* < .01 or *I* ^2^ > 50%. When there was no heterogeneity between studies, data could be merged using a fixed‐effect model; otherwise, a random‐effect model would be adopted. Sources of heterogeneity were traced using the SEN analysis and the meta‐regression test. Deek's funnel plot and visual Funnel plot, as well as Begg's and Egger's tests, were used to assess publication bias among studies, and the statistical significance level was set at *P* < .1. When publication bias appeared, the nonparametric trim and fill method will be applied to assess its possible effect on the meta‐analysis model.^39^

## 3 RESULTS

### 3.1 Clinical characteristics in included studies

**FIGURE 1:**
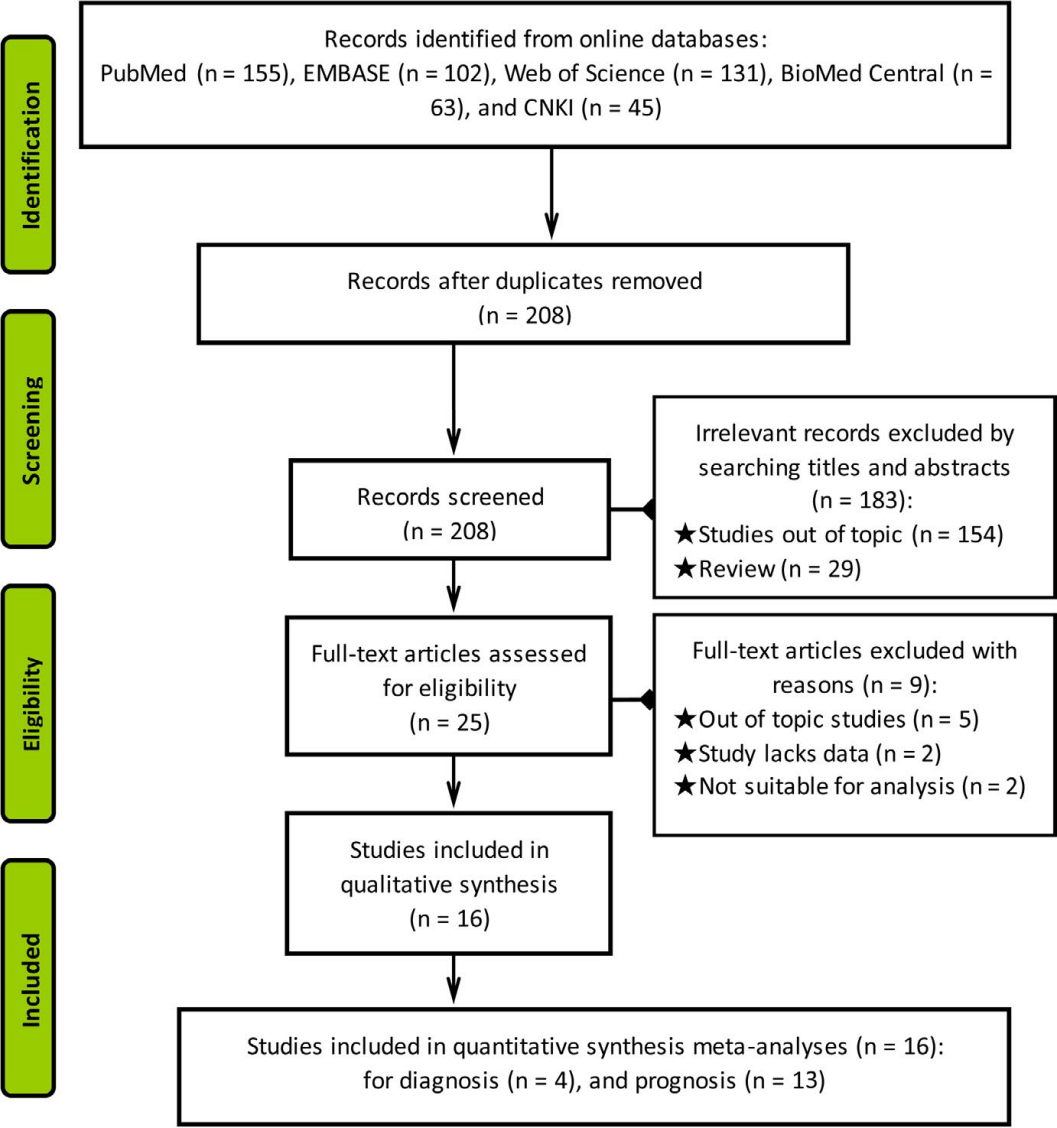
The flow chart of inclusion and exclusion processes of literature search

The inclusion and exclusion process of literature retrieval was depicted in Figure 1. As a result of database search according to the search strategy, 208 relevant studies were obtained. After carefully reading titles and abstracts, we ruled out 183 articles due to irrelevant topics or reviews, retained 25 for the full‐text evaluation, and eliminated 7 owing to a lack of data or out of topic. Of all prognostic studies, one study investigated the prognostic value of tumor‐inhibitory circRNAs on DFS,^40^ together with the other study assessed a combination of 10 circRNAs using The Cancer Genome Atlas clinical data for BC,^41^ were both eliminated. Finally, 16 articles,^20^, ^21^, ^22^, ^23^, ^24^, ^25^, ^26^, ^27^, ^28^, ^29^, ^30^, ^31^, ^32^, ^33^, ^34^, ^35^ including 4 individual studies on the diagnosis and 13 on the prognosis, were included for the subsequent meta‐analysis.

The basic characteristics of the enrolled subjects, 2438 BC cases and 271 controls (only for the diagnostic studies) from the 16 studies, were summarized in Tables 1 and 2. All BC subjects were pathologically confirmed, with early BC (stage 0, I, II) individuals in the diagnostic studies accounting for 73.49% (366/498). The included controls consisted of healthy controls and paracancer controls. All tissue and plasma samples were preoperatively collected before any treatment. The study participants encompassed both Asians and Caucasians. Of the 13 included prognostic studies, 8 provided HR and 95% CIs, which could be indirectly obtained by a formula or prognosis curve from another 5. However, only 2 studies referred to the follow‐up time. A total of 24 circRNA molecules were used in the studies, of which 19 oncogenic circRNAs were up‐regulated in BC, and 5 tumor‐inhibitory ones were down‐regulated. All circRNA expression levels were detected by quantitative reverse transcription‐polymerase chain reaction with reduced glyceraldehyde‐phosphate dehydrogenase, β‐actin, or U6 as internal reference genes.

**TABLE 1:** Clinical characteristics of BC patients in diagnostic studies on circRNAs

Study	Sample size		Control source	CircRNA type	Test method/ References	CircRNA level	Cutoff value	TP	FP	FN	TN
	BC	Control									
Li Y 2019^23^	350 (0‐II: 266)	163	Paired adjacent tissue	Circ‐VRK1	qRT‐PCR/2^−△△Ct^ method/GAPDH	Down‐regulated	0.425	217	34	133	129
Yang R 2019^32^	40 (0‐II: 33）	40	Healthy individuals	circAGFG1	qRT‐PCR/2^−△△Ct^ method/GAPDH	Up‐regulated	Unclear	26	4	14	36
Yin WB 2018^33^	20	17	Healthy individuals	hsa_circ_0001785	qRT‐PCR/2^−△△Ct^ method/GAPDH	Up‐regulated	0.981	16	4	4	13
Yin WB 2018^33^	20	17	Healthy individuals	hsa_circ_0108942	qRT‐PCR/2^−△△Ct^ method/GAPDH	Up‐regulated	0.981	16	8	4	9
Yin WB 2018^33^	20	17	Healthy individuals	hsa_circ_0068033	qRT‐PCR/2^−△△Ct^ method/GAPDH	Down‐regulated	0.981	15	7	5	10
Yin WB 2018^33^	57 (0‐II: 21)	17	Healthy individuals	hsa_circ_0001785	qRT‐PCR/2^−△△Ct^ method/GAPDH	Up‐regulated	0.981	44	5	13	12
Lü L 2017^25^	51 (0‐II: 46)	51	Adjacent noncancer tissue	hsa_circ_103110	qRT‐PCR/ΔCt method GAPDH	Down‐regulated	8.97	32	19	19	32
Lü L 2017^25^	51(0‐II: 46)	51	Adjacent noncancer tissue	hsa_circ_104689	qRT‐PCR/ΔCt method GAPDH	Down‐regulated	7.67	29	23	22	28
Lü L 2017^25^	51(0‐II: 46)	51	Adjacent noncancer tissue	hsa_circ_104821	qRT‐PCR/ΔCt method GAPDH	Down‐regulated	6.04	29	22	22	29
Lü L 2017^25^	51(0‐II: 46)	51	Adjacent noncancer tissue	hsa_circ_006054	qRT‐PCR/ΔCt method GAPDH	Up‐regulated	14.84	33	16	18	35
Lü L 2017^25^	51(0‐II: 46)	51	Adjacent noncancer tissue	hsa_circ_100219	qRT‐PCR/ΔCt method GAPDH	Up‐regulated	8.95	35	15	16	36
Lü L 2017^25^	51(0‐II: 46)	51	Adjacent noncancer tissue	hsa_circ_406697	qRT‐PCR/ΔCt method GAPDH	Up‐regulated	14.24	32	19	19	32

Abbreviations: BC, breast cancer; circRNA, circular RNA; FN, false negative; FP, false positive; GAPDH, reduced glyceraldehyde‐phosphate dehydrogenase; qRT‐PCR, quantitative reverse transcription‐polymerase chain reaction; TN, true negative; TP, true positive.

**TABLE 2:** Clinical characteristics of BC patients in prognostic studies on circRNAs

Study	CircRNA expression		CircRNA type	Expression level	Test method	Reference gene	Survival point	Follow‐up time	HR extraction
	High	Low							
Chen B 2018^20^	83	157	circEPSTI1	Up‐regualted	qRT‐PCR	β‐actin	OS, DFS	Unclear	Indirectly
Zeng K 2018^34^	82	83	circANKS1B	Up‐regualted	qRT‐PCR/2^−△△Ct^ method	GAPDH	OS	Mentioned the follow‐up process	Directly
He R 2017^22^	119	103	circGFRA1	Up‐regualted	qRT‐PCR/2^−△△Ct^ method	β‐actin	OS, DFS	Unclear	Indirectly
Yang L 2019^31^	29	28	Circ_0103552	Up‐regualted	qRT‐PCR/ΔCt	Unclear	OS	Unclear	Directly
Uhr K 2018^27^	Total: 345		CDR1‐AS	Up‐regualted	qRT‐PCR/2^−△△Ct^ method	Unclear	OS, PFS	Median: 91 mo	Directly
Xu JH 2019^29^	75	70	has_circ_001569	Up‐regualted	qRT‐PCR/2^−△△Ct^ method	GAPDH	OS	Unclear	Directly
Liu Z 2019^24^	38	98	hsa_circ_001783	Up‐regualted	qRT‐PCR	β‐actin	OS	Unclear	Directly
Zhou H 2019^35^	85	65	circFBXL5	Up‐regualted	Microarray analysis	/	OS	Unclear	Indirectly
Xu Y 2018^30^	41	35	circ_0005230	Up‐regualted	qRT‐PCR/2^−△△Ct^ method	GAPDH	OS	Unclear	Directly
Yang R 2019^32^	20	20	circAGFG1	Up‐regualted	qRT‐PCR	Unclear	OS	Unclear	Directly
Wang S 2018^28^	39	39	circ‐UBAP2	Up‐regualted	qRT‐PCR/2^−△△Ct^ method	GAPDH, U6	OS	Unclear	Indirectly
Gao D 2019^21^	49	48	circ_0006528	Up‐regualted	qRT‐PCR/2^−△△Ct^ method	Unclear	OS, RFS	Unclear	Indirectly
Smid M 2019^26^	Total: 348		circCNOT2	Up‐regualted	qRT‐PCR/2^−△△Ct^ method	Unclear	PFS	Unclear	Directly

Abbreviations: DFS, disease‐free survival; GAPDH, reduced glyceraldehyde‐phosphate dehydrogenase; HR, hazard ratio; OS, overall survival; PFS, progression‐free survival; qRT‐PCR, quantitative reverse transcription‐polymerase chain reaction; RFS, relapse‐free survival.

### 3.2 Risk assessment for heterogeneity and quality

Spearman's correlation coefficients showed that the effect size of the overall combination corresponds to *P* = .139, suggesting that there was no heterogeneity caused by threshold effects between studies. Cochran's *Q* and *I* ^2^ tests for nonthreshold effects showed a *P* = .001 and an *I* ^2^ of 83.17%, indicating significant heterogeneity among studies.

Diagnostic studies were analyzed using the QUADAS‐2 tool for a risk of bias assessment, and it was found that the QUADAS‐2 scores of all 6 studies were higher than 4 points, suggesting the high quality of the included studies (Table 3). Besides, all included case‐control studies revealed high NOS scores of over 6 points, which could be defined as high quality (Table 4).

**TABLE 3:** Study bias and quality assessment of diagnostic studies using the QUADAS‐2 checklist

Study	Risk of bias				Concerns regarding applicability			Total rated scores
	Patient selection	Index test	Reference standard	Flow and timing	Patient selection	Index test	Reference standard	
Li Y 2019^23^	Low	Low	Low	Unclear	Low	Low	Low	6
Yang R 2019^32^	Low	Unclear	Low	Unclear	Low	Unclear	Low	4
Yin WB 2018^33^	Low	Low	Low	Unclear	Low	Low	Low	6
Lü L 2017^25^	Low	Low	Low	Low	Low	Low	Low	6

Abbreviation: QUADAS, Quality Assessment for Studies of Diagnostic Accuracy.

**TABLE 4:** Study bias and quality assessment of case‐control studies using the Newcastle Ottawa Scale checklist

Study	Cohort selection				Comparability of cases and controls on the basis of the design or analysis	Outcome ascertainment			Total rated scores
	Representativeness of the exposed cohort	Selection of the nonexposed cohort	Ascertainment of exposure	Demonstration that outcome of interest was Not present at start of study		Assessment of outcome	Was follow‐up long enough for outcomes to occur	Adequacy of follow‐up of cohorts	
Chen B 2018^20^	1	1	1	1	1	1	0	0	6
Zeng K 2018^34^	1	1	1	1	1	1	1	1	8
He R 2017^22^	1	1	1	1	1	1	0	0	6
Yang L 2019^31^	1	1	1	1	1	1	0	0	6
Uhr K 2018^27^	1	1	1	1	1	1	1	1	8
Xu JH 2019^29^	1	1	1	1	1	1	0	0	6
Liu Z 2019^24^	1	1	1	1	1	1	0	0	6
Zhou H 2019^35^	1	1	1	1	1	1	0	0	6
Xu Y 2018^30^	1	1	1	1	1	1	0	0	6
Yang R 2019^32^	1	1	1	1	1	1	0	0	6
Wang S 2018^28^	1	1	1	1	1	1	0	0	6
Gao D 2019^21^	1	1	1	1	1	1	0	0	6
Smid M 2019^26^	1	1	1	1	1	1	0	0	6

### 3.3 Diagnostic performances of circRNAs

**FIGURE 2:**
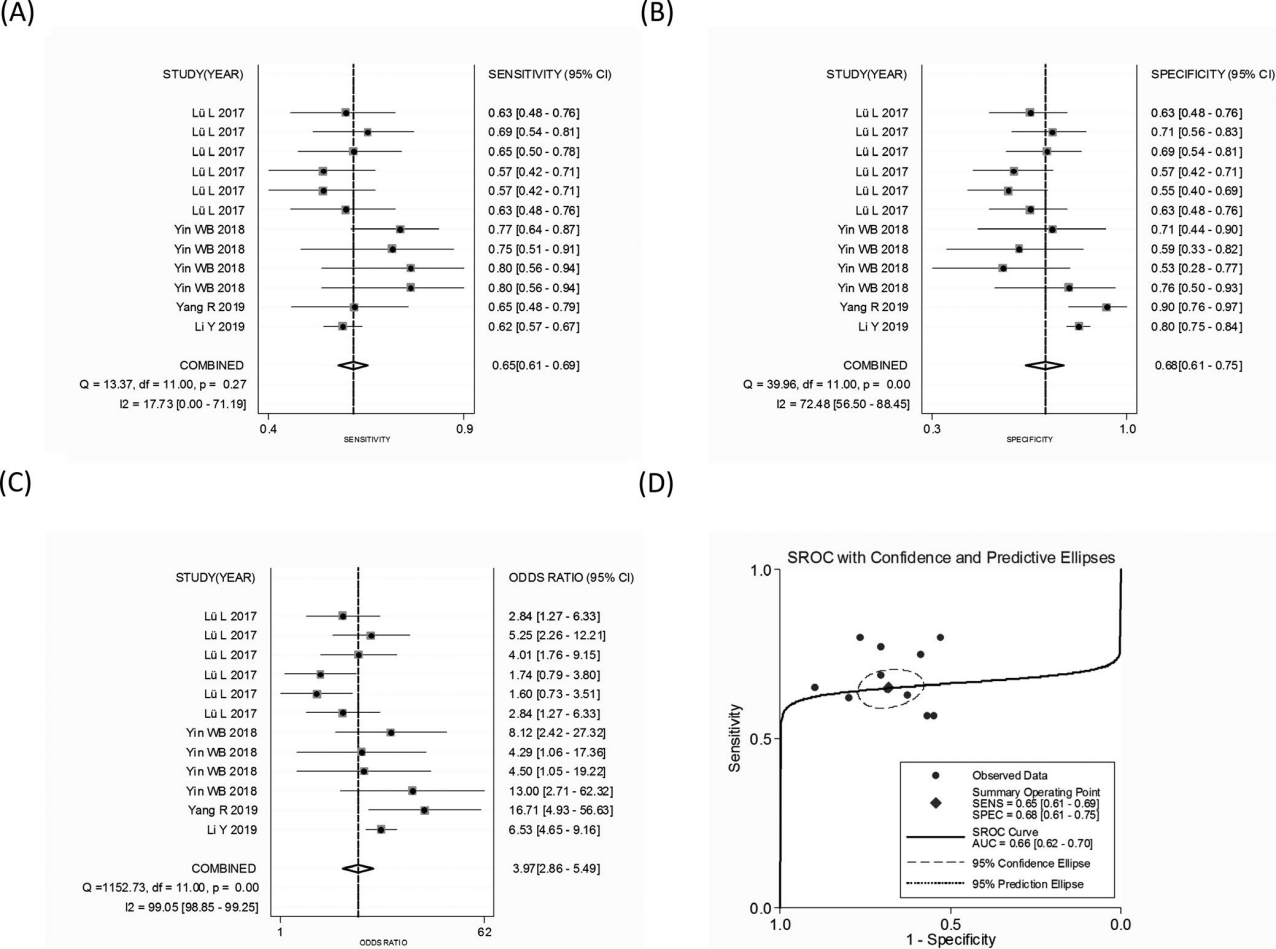
The combined (A) sensitivity, (B) specificity, (C) diagnostic odds ratio, and (D) area under the curve of abnormally expressed circular RNAs in the diagnosis of breast cancer

The overall SEN, SPE, PLR, NLR, and DOR were 0.84 (95% CI: 0.78‐0.88), 0.83 (95% CI: 0.78‐0.87), 4.95 (95% CI: 3.87‐6.33), 0.20 (95% CI: 0.15‐0.26), and 25.27 (95% CI: 17.31‐36.88), respectively, with a corresponding area under the curve (AUC) of 0.90. The forest maps of combined SEN, SPE, DOR, and AUC of circRNAs for the diagnosis of BC (including precancerous lesions; early stages 0‐II accounting for 73.49%) were shown in Figure 2.

The subgroup analysis revealed that circRNA profiling yielded a high diagnostic efficacy in distinguishing BC from healthy individuals than that from adjacent noncancer tissues (AUC: 0.81 vs 0.65). Moreover, oncogenic circRNAs also achieved a diagnostic performance higher than tumor‐inhibitory circRNAs (AUC: 0.76 vs 0.65) (Table 5).

**TABLE 5:** Stratified study of the diagnostic efficacy of circRNA profiling in BC

Variables	SEN(95% CI)	SPE (95% CI)	PLR (95% CI)	NLR (95% CI)	DOR (95% CI)	AUC
Control type						
BC vs Healthy individuals	0.75 (0.67‐0.81)	0.74 (0.65‐0.82)	2.57 (1.62‐4.08)	0.36 (0.27‐0.48)	8.33 (4.55‐15.23)	0.81
BC vs Adjacent noncancer control	0.62 (0.58‐0.66)	0.72 (0.68‐0.75)	1.86 (1.37‐2.51)	0.57 (0.48‐0.67)	3.27 (2.04‐5.26)	0.65
Function of circRNA						
Oncogenic circRNAs	0.70 (0.64‐0.75)	0.71 (0.65‐0.77)	2.26 (1.72‐2.97)	0.45 (0.37‐0.54)	5.54 (3.50‐8.76)	0.76
Tumor‐inhibitory circRNAs	0.62 (0.57‐0.66)	0.73 (0.69‐0.77)	1.76 (1.14‐2.71)	0.60 (0.46‐0.77)	2.99 (1.49‐5.99)	0.65

Abbreviations: AUC, area under the curve; BC, breast cancer; DOR, diagnostic odds ratio; NLR, negative likelihood ratio; PLR, positive likelihood ratio; SEN, sensitivity; SPE, specificity.

### 3.4 Prognostic value

**FIGURE 3:**
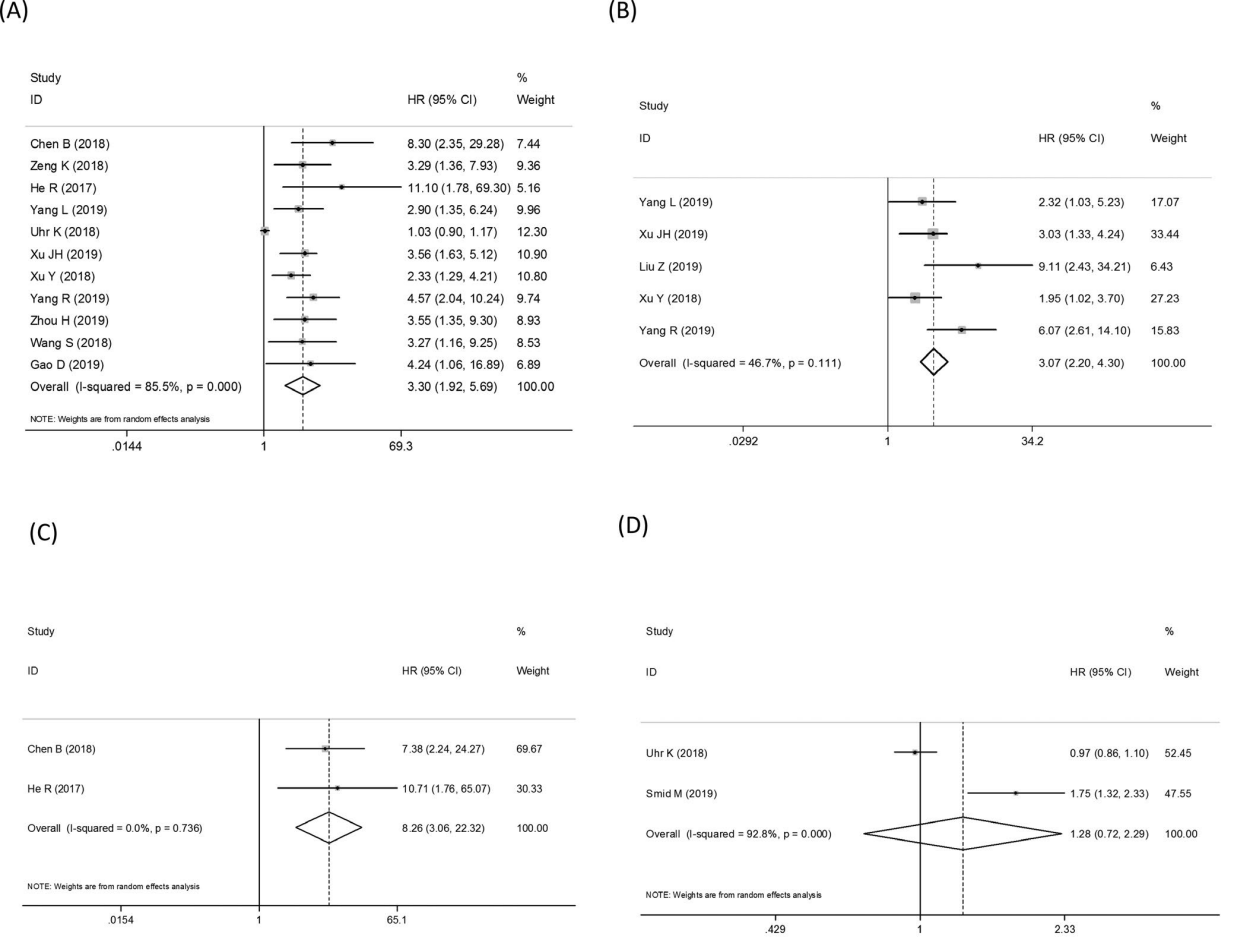
The combined hazard ratios (HRs) and 95% CIs of oncogenic circular RNAs (circRNAs) in predicting overall survival using (A) the univariate analysis and (B) multivariate analysis. The combined (C) disease‐free survival and progression‐free survival of oncogenic circRNAs

**FIGURE 4:**
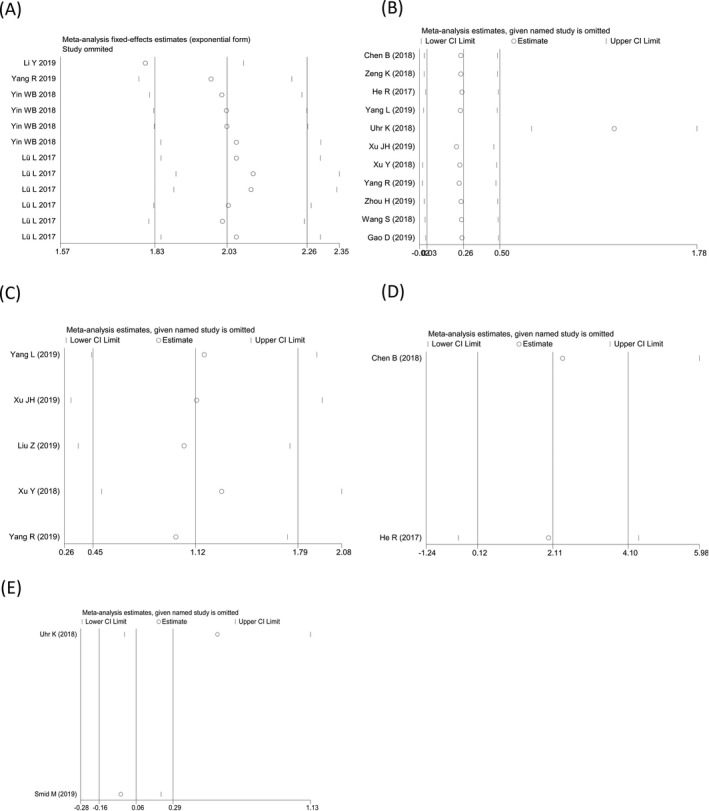
Sensitivity analyses of (A) the overall diagnostic effect, and the prognostic meta‐analyses including (B) the univariate analysis and (C) multivariate analysis of oncogenic circular RNAs (circRNAs) in predicting overall survival as well as the combined (D) disease‐free survival and (E) progression‐free survival of oncogenic circRNAs

For the prognostic analysis, summary HRs and 95% CIs were estimated using a random‐effect model. Our results showed that high expression levels of oncogenic circRNAs were significantly associated with poor OS (univariate analysis: HR = 3.30, 95% CI: 1.92‐5.69, *P* = .000, *I* ^2^ = 85.5%; multivariate analysis: HR = 3.07, 95% CI: 2.20‐4.30, *P* = .000, *I* ^2^ = 46.7%), and DFS (HR = 8.26, 95% CI: 3.06‐22.32 *P* = .000, *I* ^2^ = 0.0%) in patients with BC (Figure 3). This indicated a potential role of oncogenic circRNAs in predicting BC survival. However, the combined HR for PFS was not significant (HR = 1.28, 95% CI: 0.72‐2.29 *P* = .396, *I* ^2^ = 92.8%), and only 2 studies that, respectively, investigated the tumor‐inhibitory circRNA in PFS and DFS were enrolled in our study; the accuracy of the combined effects was therefore limited (Figure 4).

### 3.5 Sensitivity analysis and meta‐regression test

**FIGURE 5:**
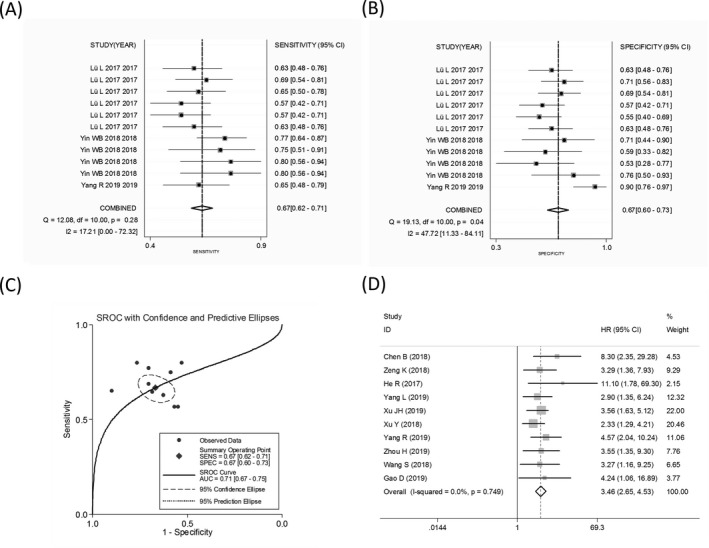
The combined (A) sensitivity, (B) specificity, and (C) area under the curve of abnormally expressed circular RNAs (circRNAs) in the diagnosis of breast cancer after an elimination of the outliers. D, The univariate analysis oncogenic circRNAs in predicting overall survival following outlier elimination

Sensitivity analysis was conducted to explore the sources of heterogeneity among studies, and outliers were found in the diagnostic meta‐analysis as well as the prognostic meta‐analyses of the OS (univariate analysis) and PFS (Figure 3). After an elimination of the outliers, the pooled SEN increased to 0.67, SPE decreased to 0.67, and AUC increased to 0.71 (Figure 5A‐C); importantly, the *I* ^2^ increased to 0% along with a P value of Cochran's *Q* test elevated to 0.343. For the prognostic effect, the pooled HR of univariate analysis in predicting OS altered to 3.46 (Figure 5D), and *I* ^2^ increased to 0% after an exclusion of the outlier. In addition, the meta‐regression was performed for analyzing the effects resulting from control type, number of cases, number of controls, and study quality. We found that the mentioned factors were not the underlying sources of heterogeneity among studies (all with *P* > .05) (Table 6).

**TABLE 6:** The underlying causes of heterogeneity of the diagnostic meta‐analysis by meta‐regression test

Meta‐regression variables	PDOR (95% CI)	*P* value
BC case number (≥100 vs <100)	0.89 (0.22‐3.58)	.8577
Control number (≥100 vs <100)	0.75 (0.10‐5.53)	.7362
Control type (healthy control vs adjacent noncancer tissue)	3.96 (0.69‐22.84)	.1032
CircRNA expression level (increased vs decreased)	1.72 (0.77‐3.83)	.1504
Study quality (QUADAS score ≥4 vs <4)	1.96 (0.17‐22.90)	.5281

Abbreviations: BC, breast cancer; PDOR, pooled diagnostic odds ratio; QUADAS, Quality Assessment for Studies of Diagnostic Accuracy.

### 3.6 Publication bias

**FIGURE 6:**
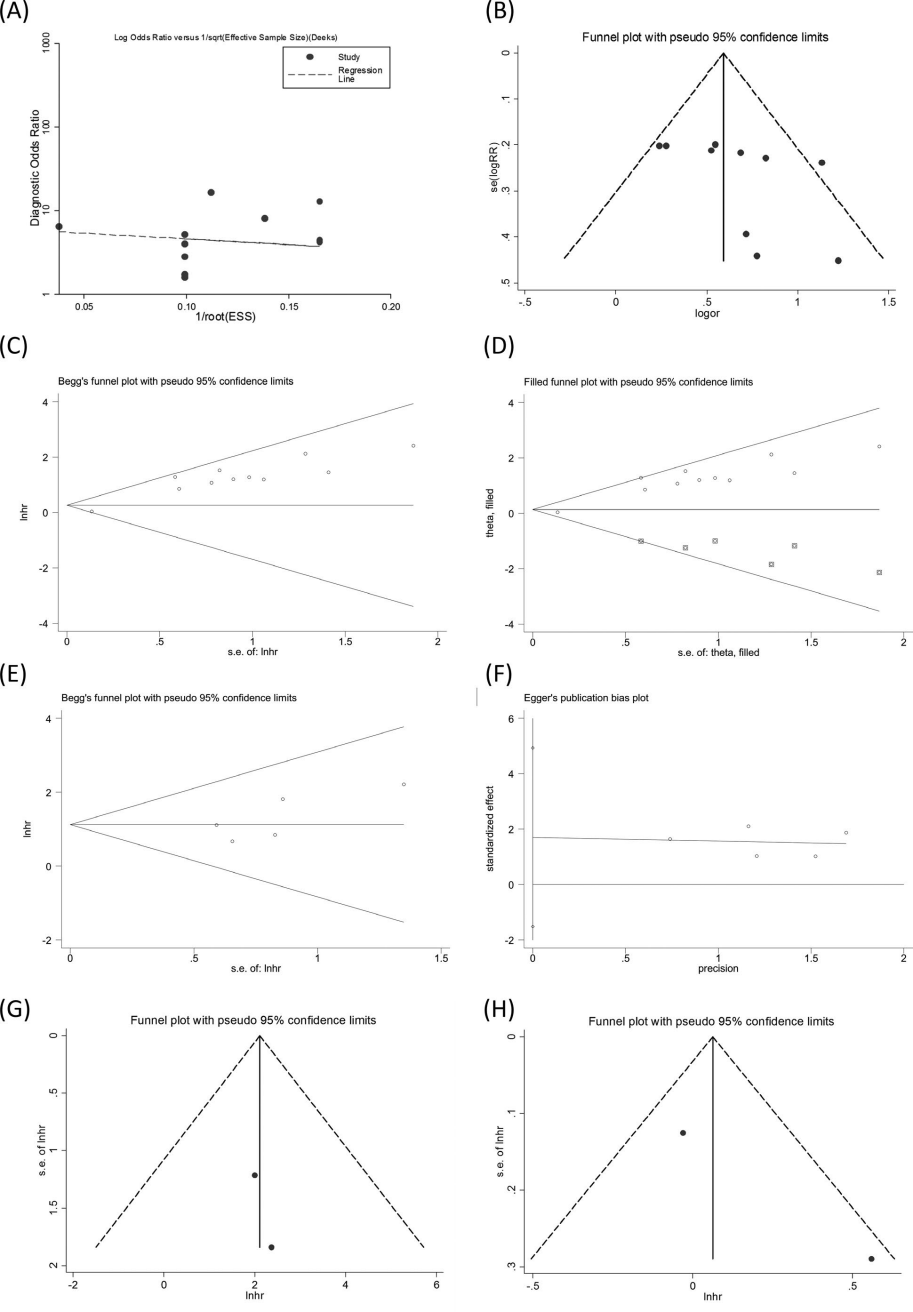
Publication bias in the diagnostic meta‐analysis evaluated by (A) Deek's funnel plot (*P* = .32) and (B) visual Funnel plot. The prognostic meta‐analyses of the univariate analysis assessed by (C) Begg's test, and (D) nonparametric trim and fill method, and the multivariate analysis by (E) Begg's and (F) Egger's tests. The judgment of publication bias in combined (G) disease‐free survival and (H) progression‐free survival using visual Funnel plot

No publication bias was observed in the pooled effects except the prognostic meta‐analysis of the OS (univariate analysis) (Figure 6). The nonparametric trim and fill method was applied to assess the possible effect of publication bias on the meta‐analysis model.^39^ The imputed data generated a symmetrical funnel plot (Figure 6D). However, the pooled effect incorporating the hypothetical data altered little from the unadjusted ones (variance = 0.367, *P* = .013 vs variance = 0.440, *P* = .016), hinting that the combined effect is not subject to the impact of publication bias.

## 4 DISCUSSION

Breast tumor malignancy that originates from mammary epithelial cells more rapidly occurs in the younger age group.^1^, ^2^ Screening and developing novel and noninvasive biomarkers will facilitate early identification and prognostic prediction of BC. CircRNAs have been proven to widely exist in many eukaryotic organisms and are mainly located in the cytoplasm or can be stored in exosomes.^10^, ^11^, ^12^, ^13^ They are not affected by exonucleases, and their expressions are more stable and difficult to degrade.^12^, ^13^ At present, there is a lack of evidence‐based medical supports for the diagnostic and prognostic value of circRNAs in BC. This study has analyzed the efficacy of circRNAs in diagnosing and predicting the prognosis of BC by a quantitative meta‐analysis.

Previous meta‐analyses have revealed that the diagnostic AUCs of circRNAs in gastric cancer (GC),^42^ colorectal cancer (CRC),^43^ hepatocellular carcinoma (HCC),^44^ and non‐small‐cell lung cancer (NSCLC) ^45^ reach 0.78, 0.79, 0.86, and 0.86, respectively, with a SEN, SPE, and AUC of circRNAs of 0.72, 0.74, and 0.79 in all malignancies.^46^ In our analysis, a total of 2438 BC patients (73.49% of stage 0, I, and II in the diagnostic studies) were included. Our results showed that circRNAs presented high diagnostic value for BC, with a SEN and SPE of 0.65 and 0.68, respectively, and the corresponding AUC of 0.66. The ratio of TP to FP (DOR) in diagnostic studies is another important indicator for evaluating the effectiveness of circRNA profiling.^47^ The higher the value is, the better the efficiency of the diagnostic test will be. A DOR value of less than 1 indicates low diagnostic efficiency of a test. In our study, the DOR of circRNA profiling to diagnose BC was 3.97, suggesting a relatively high diagnostic performance of this test. In addition, the combined PLR of 2.04 indicates that the probability of positive results of circRNA profiling in BC patients is 2 times higher than that in controls. The combined NLR was 0.51. This indicates that only 51% of negative results of circRNA profiling are FN. The data above fully prove that circRNA detection can be an effective method for early BC‐assisted diagnosis. The subgroup analyses reveal that oncogenic circRNAs are more effective than tumor‐inhibitory circRNAs in the diagnosis of BC, as with their AUCs. We consider that this can be related to the kurtosis of circRNA expression in BC. The expressions of oncogenic circRNAs are up‐regulated in BC, and the high expression peak in newly diagnosed patients is more conducive to detection. Moreover, we have found that circRNAs yield higher accuracy in differentiating BC from healthy individuals than that from adjacent noncancerous controls. Nonetheless, the number of samples included in the subgroup analysis has curtailed compared with that in the whole analysis, and the conclusion needs to be confirmed by more large sample size studies in the future.

Circular RNAs has been reported to be associated with the prognosis of multiple malignancies.^48^, ^49^ At present, studies have systematically evaluated the prognostic efficacy of circRNA profiling in CRC,^43^ HCC,^44^ and NSCLC^45^ and have shown that the higher the expression levels of oncogenic circRNAs are, the worse the prognosis of cancer patients will be, whereas the survival rate of tumor patients with overexpressions of tumor‐inhibited circRNAs is significantly higher than that of those with low expressions. In this regard, biofunctions of different circRNAs are distinct in malignant tumors. We have further evaluated the efficacy of circRNAs in monitoring the prognosis of BC based on the different biofunctions of circRNAs and have divided the expression profiles of circRNAs into oncogenic and tumor‐inhibitory groups. The survival analysis shows that BC patients with low oncogenic circRNA levels present significantly prolonged OS and DFS, while those with the low expressions of tumor‐inhibitory circRNAs show significantly decreased OS compared with the cases of high expressions of anti‐cancer circRNAs. This suggests that these circRNA molecules exhibit promising efficacy in prognostic evaluation and monitoring of BC.

The generation of heterogeneity is inevitable in the process of a meta‐analysis, and its main sources consist of threshold and nonthreshold effects.^50^ Spearman's correlation coefficients show that consolidated statistics and heterogeneity in subgroup analyses chiefly result from threshold effects that can be affected by various thresholds or cutoff values. The cutoff values and internal reference genes used for the relative quantification of circRNA included in this study are presumed to be one of the main causes of heterogeneity. Moreover, we have also explored the possible factors bringing about heterogeneity using the SEN analysis and the meta‐regression test. Deviant outliers have been found in the SEN analysis, and an elimination of them could alter the heterogeneity of the pooled effects, suggesting that included deviant outliers are major causes of heterogeneity. The meta‐regression has traced the factors, such as control type, number of cases, and study quality, and has revealed that the mentioned factors are not likely to be sources of heterogeneity between the studies.

Nevertheless, some limitations still remain in our study. Firstly, the combination of study effect sizes is predominantly based on the Chinese population, so the underlying population bias may exist. Secondly, the types of included circRNAs and the samples are not unified, and this can be the source of heterogeneity between the included studies. Thirdly, the included studies on evaluating the diagnostic efficacy of circRNA in BC as well as their performance in predicting DFS and PFS are all limited, so the relevant meta‐analysis is unavailable.

## 5 CONCLUSIONS

In summary, circRNAs can be used as prominent auxiliary indicators for the diagnosis and prognosis evaluation of BC. However, the conclusion of this study still needs to be confirmed by more high‐quality studies with large samples.

## CONFLICT OF INTEREST

None declared.

## References

[1] Woolston C . Breast cancer. Nature. 2015;527:S101. 26580154 10.1038/527S101a

[2] Ward EM , DeSantis CE , Lin CC , et al. Cancer statistics: breast cancer in situ. CA Cancer J Clin. 2015;65:481‐495. 26431342 10.3322/caac.21321

[3] Chen W , Zheng R , Baade PD , et al. Cancer statistics in China, 2015. CA Cancer J Clin. 2016;66:115‐132. 26808342 10.3322/caac.21338

[4] Momenimovahed Z , Salehiniya H . Epidemiological characteristics of and risk factors for breast cancer in the world. Breast Cancer (Dove Med Press). 2019;11:151‐164. 31040712 10.2147/BCTT.S176070 PMC6462164

[5] Rey‐Vargas L , Sanabria‐Salas MC , Fejerman L , Serrano‐Gómez SJ . Risk factors for triple‐negative breast cancer among Latina women. Cancer Epidemiol Biomarkers Prev. 2019;28:1771‐1783. 31455670 10.1158/1055-9965.EPI-19-0035

[6] Coughlin SS . Social determinants of breast cancer risk, stage, and survival. Breast Cancer Res Treat. 2019;177:537‐548. 31270761 10.1007/s10549-019-05340-7

[7] Alimirzaie S , Bagherzadeh M , Akbari MR . Liquid biopsy in breast cancer: a comprehensive review. Clin Genet. 2019;95:643‐660. 30671931 10.1111/cge.13514

[8] Lima ZS , Ebadi MR , Amjad G , Younesi L . Application of imaging technologies in breast cancer detection: a review article. Open Access Maced J Med Sci. 2019;7:838‐848. 30962849 10.3889/oamjms.2019.171 PMC6447343

[9] Tomlinson IP , Whyman A , Barrett JA , Kremer JK . Tumour marker CA15‐3: possible uses in the routine management of breast cancer. Eur J Cancer. 1995;31A:899‐902. 7646918 10.1016/0959-8049(94)00447-1

[10] Qu S , Liu Z , Yang X , et al. The emerging functions and roles of circular RNAs in cancer. Cancer Lett. 2018;414:301‐309. 29174799 10.1016/j.canlet.2017.11.022

[11] Ng WL , Mohd Mohidin TB , Shukla K . Functional role of circular RNAs in cancer development and progression. RNA Biol. 2018;15:995‐1005. 29954251 10.1080/15476286.2018.1486659 PMC6259826

[12] Ebbesen KK , Hansen TB , Kjems J . Insights into circular RNA biology. RNA Biol. 2017;14:1035‐1045. 27982727 10.1080/15476286.2016.1271524 PMC5680708

[13] Zhang Z , Yang T , Xiao J . Circular RNAs: promising biomarkers for human diseases. EBioMedicine. 2018;34:267‐274. 30078734 10.1016/j.ebiom.2018.07.036 PMC6116471

[14] Chivukula RR , Mendell JT . Circular reasoning: microRNAs and cell‐cycle control. Trends Biochem Sci. 2008;33:474‐481. 18774719 10.1016/j.tibs.2008.06.008 PMC2824243

[15] Fischer JW , Leung AKL . CircRNAs: a regulator of cellular stress. Crit Rev Biochem Mol Biol. 2017;52:220‐233. 28095716 10.1080/10409238.2016.1276882 PMC5526226

[16] Li YF , Zhang J , Yu L . Circular RNAs regulate cancer onset and progression via Wnt/β‐catenin signaling pathway. Yonsei Med J. 2019;60:1117‐1128. 31769242 10.3349/ymj.2019.60.12.1117 PMC6881706

[17] Zhang Q , Wang W , Zhou Q , et al. Roles of circRNAs in the tumour microenvironment. Mol Cancer. 2020;19:14. 31973726 10.1186/s12943-019-1125-9 PMC6977266

[18] Meng S , Zhou H , Feng Z , et al. CircRNA: functions and properties of a novel potential biomarker for cancer. Mol Cancer. 2017;16:94. 28535767 10.1186/s12943-017-0663-2 PMC5440908

[19] Zhang HD , Jiang LH , Sun DW , Hou JC , Ji ZL . CircRNA: a novel type of biomarker for cancer. Breast Cancer. 2018;25:1‐7. 28721656 10.1007/s12282-017-0793-9

[20] Chen BO , Wei W , Huang X , et al. circEPSTI1 as a prognostic marker and mediator of triple‐negative breast cancer progression. Theranostics. 2018;8:4003‐4015. 30083277 10.7150/thno.24106 PMC6071524

[21] Gao D , Qi X , Zhang X , Fang K , Guo Z , Li L . hsa_circRNA_0006528 as a competing endogenous RNA promotes human breast cancer progression by sponging miR‐7‐5p and activating the MAPK/ERK signaling pathway. Mol Carcinog. 2019;58:554‐564. 30520151 10.1002/mc.22950

[22] He R , Liu P , Xie X , et al. circGFRA1 and GFRA1 act as ceRNAs in triple negative breast cancer by regulating miR‐34a. J Exp Clin Cancer Res. 2017;36:145. 29037220 10.1186/s13046-017-0614-1 PMC5644184

[23] Li Y , Li H . Circular RNA VRK1 correlates with favourable prognosis, inhibits cell proliferation but promotes apoptosis in breast cancer. J Clin Lab Anal. 2020;34:e22980. 31970831 10.1002/jcla.22980 PMC6977307

[24] Liu Z , Zhou Y , Liang G , et al. Circular RNA hsa_circ_001783 regulates breast cancer progression via sponging miR‐200c‐3p. Cell Death Dis. 2019;10:55. 30670688 10.1038/s41419-018-1287-1 PMC6343010

[25] Lü L , Sun J , Shi P , et al. Identification of circular RNAs as a promising new class of diagnostic biomarkers for human breast cancer. Oncotarget. 2017;8:44096‐44107. 28484086 10.18632/oncotarget.17307 PMC5546465

[26] Smid M , Wilting SM , Uhr K , et al. The circular RNome of primary breast cancer. Genome Res. 2019;29:356‐366. 30692147 10.1101/gr.238121.118 PMC6396421

[27] Uhr K , Sieuwerts AM , de Weerd V , et al. Association of microRNA‐7 and its binding partner CDR1‐AS with the prognosis and prediction of 1(st)‐line tamoxifen therapy in breast cancer. Sci Rep. 2018;8:9657. 29941867 10.1038/s41598-018-27987-w PMC6018428

[28] Wang S , Li Q , Wang Y , et al. Upregulation of circ‐UBAP2 predicts poor prognosis and promotes triple‐negative breast cancer progression through the miR‐661/MTA1 pathway. Biochem Biophys Res Commun. 2018;505:996‐1002. 30314706 10.1016/j.bbrc.2018.10.026

[29] Xu J‐H , Wang Y , Xu D . Hsa_circ_001569 is an unfavorable prognostic factor and promotes cell proliferation and metastasis by modulating PI3K‐AKT pathway in breast cancer. Cancer Biomark. 2019;25:193‐201. 31104012 10.3233/CBM-182293 PMC13082401

[30] Xu YI , Yao Y , Leng K , et al. Increased expression of circular RNA circ_0005230 indicates dismal prognosis in breast cancer and regulates cell proliferation and invasion via miR‐618/CBX8 signal pathway. Cell Physiol Biochem. 2018;51:1710‐1722. 30504704 10.1159/000495675

[31] Yang L , Song C , Chen Y , Jing G , Sun J . Circular RNA circ_0103552 forecasts dismal prognosis and promotes breast cancer cell proliferation and invasion by sponging miR‐1236. J Cell Biochem. 2019;120:15553‐15560. 31056795 10.1002/jcb.28822

[32] Yang R , Xing L , Zheng X , Sun Y , Wang X , Chen J . The circRNA circAGFG1 acts as a sponge of miR‐195‐5p to promote triple‐negative breast cancer progression through regulating CCNE1 expression. Mol Cancer. 2019;18:4. 30621700 10.1186/s12943-018-0933-7 PMC6325825

[33] Yin W‐B , Yan M‐G , Fang X , Guo J‐J , Xiong W , Zhang R‐P . Circulating circular RNA hsa_circ_0001785 acts as a diagnostic biomarker for breast cancer detection. Clin Chim Acta. 2018;487:363‐368. 29045858 10.1016/j.cca.2017.10.011

[34] Zeng K , He B , Yang BB , et al. The pro‐metastasis effect of circANKS1B in breast cancer. Mol Cancer. 2018;17:160. 30454010 10.1186/s12943-018-0914-x PMC6240936

[35] Zhou H , Tang G , Zhao MI , et al. circFBXL5 promotes breast cancer progression by sponging miR‐660. J Cell Mol Med. 2020;24:356‐361. 31729134 10.1111/jcmm.14737 PMC6933392

[36] Moher D , Liberati A , Tetzlaff J , Altman DG ; PRISMA Group . Preferred reporting items for systematic reviews and meta‐analyses: the PRISMA statement. PLoS Med. 2009;6:e1000097. 19621072 10.1371/journal.pmed.1000097 PMC2707599

[37] Whiting PF , Rutjes AWS , Westwood ME , et al. QUADAS‐2: a revised tool for the quality assessment of diagnostic accuracy studies. Ann Intern Med. 2011;155:529‐536. 22007046 10.7326/0003-4819-155-8-201110180-00009

[38] Stang A . Critical evaluation of the Newcastle‐Ottawa scale for the assessment of the quality of nonrandomized studies in meta‐analyses. Eur J Epidemiol. 2010;25:603‐605. 20652370 10.1007/s10654-010-9491-z

[39] Duval S , Tweedie R . Trim and fill: a simple funnel‐plot‐based method of testing and adjusting for publication bias in meta‐analysis. Biometrics. 2000;56:455‐463. 10877304 10.1111/j.0006-341x.2000.00455.x

[40] Zhou S‐Y , Chen W , Yang S‐J , et al. Circular RNA circVAPA regulates breast cancer cell migration and invasion via sponging miR‐130a‐5p. Epigenomics. 2020;12:303‐317. 31920104 10.2217/epi-2019-0124

[41] Deng L , Zhang W , Shi Y , Tang Y . Fusion of multiple heterogeneous networks for predicting circRNA‐disease associations. Sci Rep. 2019;9:9605. 31270357 10.1038/s41598-019-45954-x PMC6610109

[42] Lin Y , Luo X , Cui Z , Chen Y . Diagnostic potential for circular RNAs in gastric carcinoma: a meta‐analysis. Clin Lab. 2019;65 10.7754/Clin.Lab.2018.180810 30868864

[43] Li C , He X , Zhang L , Li L , Zhao W . A pair‐wise meta‐analysis highlights circular RNAs as potential biomarkers for colorectal cancer. BMC Cancer. 2019;19:957. 31615475 10.1186/s12885-019-6136-9 PMC6794748

[44] Jiang Y‐L , Shang M‐M , Dong S‐Z , Chang Y‐C . Abnormally expressed circular RNAs as novel non‐invasive biomarkers for hepatocellular carcinoma: a meta‐analysis. World J Gastrointest Oncol. 2019;11:909‐924. 31662829 10.4251/wjgo.v11.i10.909 PMC6815919

[45] Huang X , Zhang W , Shao Z . Prognostic and diagnostic significance of circRNAs expression in lung cancer. J Cell Physiol. 2019;234:18459‐18465. 30895620 10.1002/jcp.28481

[46] Wang M , Yang Y , Xu J , Bai W , Ren X , Wu H . CircRNAs as biomarkers of cancer: a meta‐analysis. BMC Cancer. 2018;18:303. 29554887 10.1186/s12885-018-4213-0 PMC5859638

[47] Cleophas TJ , Zwinderman AH . Meta‐analyses of diagnostic studies. Clin Chem Lab Med. 2009;47:1351‐1354. 19817647 10.1515/CCLM.2009.317

[48] Ding H‐X , Lv Z , Yuan Y , Xu Q . The expression of circRNAs as a promising biomarker in the diagnosis and prognosis of human cancers: a systematic review and meta‐analysis. Oncotarget. 2017;9:11824‐11836. 29545939 10.18632/oncotarget.23484 PMC5837763

[49] Huang X , Zhang Z , Qing X , et al. Dysregulated expression of circular RNAs serve as prognostic and clinicopathological markers in cancer. J Cancer. 2019;10:1825‐1832. 31205539 10.7150/jca.29438 PMC6547981

[50] Lin X , Yuan J , Zhao Y , Zha Y . Urine interleukin‐18 in prediction of acute kidney injury: a systemic review and meta‐analysis. J Nephrol. 2015;28:7‐16. 24899123 10.1007/s40620-014-0113-9 PMC4322238

